# Comparison of post-earthquake trauma and anxiety levels of intensive care unit nurses and inpatient unit nurses: a cross-sectional study

**DOI:** 10.1590/1980-220X-REEUSP-2025-0312en

**Published:** 2026-03-02

**Authors:** Zehra Kilinç, Elif Ates Budak, Hatice Arslan, Süreyya Yiğitalp Rençber

**Affiliations:** 1 Dicle University, Faculty of Medicine, Department of Public Health, Diyarbakır, Turkey.; 2 Dicle University, Medical Faculty, Department of Psychiatry, Diyarbakir, Turkey.; 3 Dicle University, Hospital Health Care Services Assistant Manager, Diyarbakır, Turkey.; 4 Batman University, Faculty of Health Sciences, Department of Public Health, Batman, Turkey.

**Keywords:** Anxiety, Earthquakes, Hospitals, Nursing Staff, Psychological Trauma, Ansiedade, Terremotos, Hospitais, Recursos Humanos de Enfermagem, Trauma Psicológico

## Abstract

**Objective:**

This study aimed to assess and compare post-earthquake trauma and anxiety in intensive care unit (ICU) and inpatient unit nurses after the Kahramanmaraş earthquake in February 2023 at Dicle University Hospital in Diyarbakır, Türkiye.

**Method:**

This cross-sectional study was conducted from July 1 to 31, 2023, with 244 nurses (89 ICU and 155 inpatient unit). A sociodemographic form, the Post-Earthquake Trauma Level Determination Scale (PETLDS), and the Beck Anxiety Inventory (BAI) were administered. Statistical analyses included the chi-square test, *t* test, Mann-Whitney *U* test, and Spearman correlation test.

**Results:**

In this study, 12.3% of all participants exhibited moderate anxiety and 13.9% displayed severe trauma. The mean scores and subscales of the PETLDS did not differ significantly between the two groups (inpatient: 48.2 ± 20.0 vs ICU: 45.6 ± 18.0; *p* > 0.05). Also, no statistically significant differences were found in the anxiety levels measured using the BAI according to the type of unit (*p* > 0.05). However, both groups presented notable proportions of psychological symptomatology.

**Conclusion:**

Both ICU and inpatient unit nurses experienced comparable and considerable levels of post-disaster trauma and anxiety. The findings underscore the need to integrate psychological support programs and resilience strategies for healthcare personnel working during and in the aftermath of disasters.

## RESUMO

**Objetivo:**

Avaliar e comparar o trauma pós-terremoto e a ansiedade em enfermeiros das unidades de terapia intensiva e de internação após o terremoto de Kahramanmaraş de fevereiro de 2023, no Hospital Universitário de Dicle, em Diyarbakır, Turquia.

**Método:**

Foi realizado um estudo transversal entre 1º e 31 de julho de 2023 com 244 enfermeiros (89 das unidades de terapia intensiva e 155 da de internação). Foi aplicado um formulário sociodemográfico, a Escala de Determinação do Nível de Trauma Pós-Terremoto (PETLDS, na sigla em inglês) e o Inventário de Ansiedade de Beck (BAI, na sigla em inglês). As análises estatísticas incluíram os testes de qui-quadrado, *t* de Student, U de Mann-Whitney e correlação de Spearman.

**Resultados:**

Do total de participantes, 12,3% apresentaram ansiedade moderada e 13,9% apresentaram trauma grave. As médias e subescalas da PETLDS não diferiram significativamente entre os dois grupos (internação: 48,2 ± 20,0 vs. UTI: 45,6 ± 18,0; p > 0,05). Também não houve diferenças estatisticamente significativas nos níveis de ansiedade avaliados pelo BAI de acordo com o tipo de unidade (p > 0,05). No entanto, ambos os grupos apresentaram proporções relevantes de sintomatologia psicológica.

**Conclusão:**

Enfermeiros das unidades de terapia intensiva e de internação vivenciaram níveis semelhantes e consideráveis de trauma e ansiedade pós-desastre. Os achados destacam a necessidade de integrar programas de apoio psicológico e estratégias de resiliência para profissionais de saúde que atuam durante e após desastres.

## INTRODUCTION

Natural disasters such as earthquakes are among the most devastating uncontrollable events, causing widespread societal and community-level impacts and resulting in significant losses^(1,2)^. An earthquake can cause extensive loss of life and physical destruction, besides social and psychological impacts^(1,3)^. The fear experienced during an earthquake, damage to homes and workplaces, and loss of loved ones can all lead to psychological stress, which can potentially trigger psychiatric disorders or exacerbate existing symptoms. Post-earthquake psychological problems commonly include anxiety, depression, panic attacks, sleep disturbances, and post-traumatic stress disorder (PTSD)^(4)^.

In disaster situations, healthcare services face challenges that differ markedly from routine operations and are often unpredictable. Hospitals must continue to deliver patient care, medical support, and institutional services without interruption, while healthcare workers fulfill vital roles in emergency response and life-saving interventions^(5)^. Nurses are the largest professional group within the continuous workforce delivering healthcare services, playing a vital role in both preparedness and rescue phases, thereby contributing to community resilience in the face of disasters^(6)^.

The work environment and sociodemographic characteristics of healthcare professionals are significant factors influencing their physical and mental health^(7)^. Intensive care units (ICUs) and emergency departments are identified as high-risk work areas in hospitals^(8)^. ICU patients often face life-threatening conditions, require close monitoring, and have higher rates of mortality and morbidity; therefore, ICU nurses must possess advanced skills and qualifications^(9)^. Literature shows that, under normal circumstances, ICU nurses report higher levels of psychological burden, such as stress and anxiety, compared with nurses in other units^(10,11,12,13,14)^. However, one study discovered no significant differences in depression, anxiety, or workplace stressors between ICU and general-ward nurses^(15)^.

The exposure of ICU nurses to traumatic events such as earthquakes, including witnessing the event, hearing details about it, or merely learning of it, can trigger PTSD symptoms such as re-experiencing, avoidance, hyperarousal, and cognitive changes^(16)^. Numerous studies have reported an increased risk of PTSD, depression, and anxiety among emergency responders and healthcare personnel following disasters. Available data also indicate that chronic exposure to high stress in various nursing units is closely linked to absenteeism and health problems, with frequent co-occurrence of anxiety and depression^(17)^. Additionally, nurses working under intense stress or witnessing trauma commonly experience burnout, anxiety, depression, and secondary traumatic stress^(18,19)^.

On February 6, 2023, two major earthquakes (Mw 7.7 and Mw 7.6) centered in Kahramanmaraş caused widespread destruction and loss of life across 11 provinces in Türkiye. Diyarbakır was one of the affected cities, with 414 deaths, 902 injuries, and severe damage to thousands of buildings^(20)^. This study aimed to assess the psychological impact, specifically trauma and anxiety levels, among ICU and regular inpatient unit nurses at a tertiary-level university hospital in Diyarbakır 5 months after the earthquakes^(21)^.

Despite increasing evidence on disaster-related mental health among healthcare workers, potential knowledge gaps remain regarding unit-specific differences in post-earthquake symptomatology and the practical implications for hospital workforce management. Evidence is inconsistent on whether ICU nurses experience a greater psychological burden than ward nurses in disaster contexts. Also, a few studies have investigated these differences within the same institution and time window post-event. Moreover, previous studies have often underemphasized how findings translate into human resources (HR) management and mental health programs in hospital settings.

This study addressed these gaps by comparing post-earthquake trauma and anxiety among ICU and inpatient unit nurses within a tertiary university hospital located in an affected province, 5 months after the February 2023 earthquakes. It focused on two high-relevance groups working under similar institutional policies and resources, providing timely, practice-oriented evidence to inform HR planning, resilience-building, and mental health policy development for nursing staff in disaster-prone regions.

## METHOD

### Study Design

This was a cross-sectional study reported in accordance with the Strengthening the Reporting of Observational Studies in Epidemiology checklist.

### Population and Sample

This analytical cross-sectional study was conducted from July 1 to 31, 2023, at Dicle University Hospital in Diyarbakır, Türkiye. The target population comprised all 450 registered nurses working in adult ICUs and general inpatient wards during the study period. A census strategy was adopted; nurses who were on leave for the entire recruitment period were excluded. A total of 244 nurses (54.2%) were included in the study after receiving detailed information about the study objective and providing verbal consent.

### Data Collection Tools

The data were gathered from the nurses’ own units through face-to-face interviews using a three-part structured questionnaire. The first part captured sociodemographic characteristics and earthquake-related experiences. The second comprised the 20-item Post-Earthquake Trauma Level Determination Scale (PETLDS; 5-point Likert scale, total score 20–100, Cronbach’s α = 0.87, and Turkish cut-off ≥ 52.4 ± 5.1 indicating clinically significant trauma). The third comprised the 21-item Beck Anxiety Inventory (BAI; 4-point Likert scale, total score 0–63, and Cronbach’s α = 0.93, with scores interpreted as: 0–7, none; 8–15, mild; 16–25, moderate; and 26–63, severe anxiety)^(22,23)^.

### Statistical Analysis

Primary outcomes were PETLDS and BAI total scores, whereas the principal exposure variable was the work setting (ICU vs ward). Prespecified potential confounders included sex,age, marital status, educational level, earthquake-related home damage, prior earthquake experience, bereavement of a close relative or friend, and personal injury. Statistical analyses were performed using IBM SPSS Statistics v24. Categorical variables were presented as frequencies and percentages, whereas continuous variables were expressed as mean ± standard deviation or median [interquartile range] according to Kolmogorov–Smirnov normality testing. Group comparisons of categorical data, normally distributed data, and non-normally distributed data were conducted using Pearson’s χ^2^ test, independent-samples *t* test, and Mann–Whitney *U* test, respectively. The correlations between continuous variables were examined with Spearman’s correlation. Two-tailed *p*-values <0.05 indicated statistically significant differences. Given the descriptive and comparative aim of this cross-sectional study, the primary analyses focused on unadjusted between-group comparisons (ICU vs ward) without causal inferences. We did not fit multivariable regression models because several candidate covariates (e.g., earthquake-related losses) were plausibly downstream of the exposure (work unit) in a post-disaster setting; routine adjustment for downstream variables may introduce bias by conditioning on mediators or colliders. Moreover, observed between-group differences were small and nonsignificant, and therefore, additional adjustment was unlikely to change the substantive conclusions while potentially encouraging over-interpretation. We emphasize estimation and transparent reporting, and outline adjusted modeling as a priority for future multicenter, longitudinal research.

### Ethics Approval and Consent

The study protocol was approved by the Dicle University Non-Interventional Clinical Research Ethics Committee (Decision No. 29, June 14, 2023). All procedures adhered to the Declaration of Helsinki. The ethics committee approved the use of informed verbal consent for this minimal-risk, questionnaire-based study. Prior to participation, all nurses received standardized information about the study purpose, procedures,risks, and benefits, and provided verbal consent to participate. Administrative permission to conduct the study within the hospital was obtained from the institutional leadership. Data were collected anonymously without personal identifiers and stored on encrypted devices, and were accessible only to the research team.

## RESULTS

Examination of the demographic and professional characteristics of the participating nurses revealed that 74.2% of the regular inpatient unit nurses and 58.4% of the ICU nurses were female. The mean age of the inpatient unit and ICU nurses was 37.0 ± 9.1 years and 31.7 ± 7.7 years, respectively. In terms of years of service, inpatient unit nurses had a mean of 15.0 ± 9.7 years, whereas ICU nurses had a mean of 9.3 ± 8.0 years; this difference was statistically significant (*p* < 0.001).

Regarding marital status, 61.3% of inpatient unit nurses and 48.3% of ICU nurses were married. Regarding educational level, 60.6% of inpatient unit nurses and 52.8% of ICU nurses held a bachelor’s degree (Table 1).

**Table 1:** Comparison of demographic and occupational characteristics of nurses working in inpatient and intensive care units - Diyarbakir,Türkiye,2023.

		Inpatient			Intensive care			*p* ^*^
		*n*		%	*n*		%
**Sex**	Female	115		74.2	52		58.4	**0.011**
	Male	40		25.8	37		41.6	
**Age, Mean ± SD**			37.0 ± 9.1			31.7 ± 7.7		< 0.001^**^
**Marital Status**	Single	60		38.7	46		51.7	**0.049**
	Married	95		61.3	43		48.3	
**Education Level**	High school	23		14.8	19		21.3	0.494
	Associate degree	26		16.8	14		15.7	
	Bachelor’s degree	94		60.6	47		52.8	
	Postgraduate degree	12		7.7	9		10.1	
**Years of employment, Mean ± SD**			15.0 ± 9.7			9.3 ± 8.0		*p* < 0.001^**^

*Chi-square test

**Mann”Whitney *U* test

Further, 82.5% of inpatient unit nurses and 83.1% of ICU nurses had previously experienced an earthquake, with no significant difference between groups (*p* = 0.676). Moreover, 92.5% of inpatient unit nurses and 98.9% of ICU nurses were working in one of the 10 provinces impacted by the earthquake at the time of the event, again without a significant group difference (*p* = 0.264). Regarding home damage, 53.5% of inpatient unit nurses reported no damage, 38.1% reported minor damage, and 8.4% reported moderate-to-severe damage or collapse; among ICU nurses, these proportions were 61.8%, 31.5%, and 6.7%, respectively (*p* = 0.456). Injuries sustained during the earthquake were reported by 2.6% of inpatient unit nurses and 4.5% of ICU nurses (*p* = 0.468). Finally, 17.4% of inpatient unit nurses and 24.7% of ICU nurses lost a close relative in the earthquake, with no significant difference between groups (*p* = 0.171) (Table 2).

**Table 2:** Department-wise comparison of earthquake-related experiences among nurses in Diyarbakir - Diyarbakir, Türkiye, 2023.

		Inpatient		Intensive care		*p* ^*^
		*n*	%	*n*	%
**Have you ever experienced an earthquake before?**	Yes	132	85.2	74	83.1	0.676
	No	23	14.8	15	16.9	
**Where were you during the earthquake?**	One of the ten cities most affected by the earthquake	148	95.5	88	98.9	0.264
	Other cities	7	4.5	1	1.1	
**Did your home sustain damage in the earthquake?**	No damage	83	53.5	55	61.8	0.456
	Slight	59	38.1	28	31.5	
	Moderate or major damage/collapse	13	8.4	6	6.7	
**Did you sustain any injuries?**	Yes	4	2.6	4	4.5	0.468
	No	151	97.4	85	95.5	
**Did you lose any relatives during the earthquake?**	Yes	27	17.4	22	24.7	0.171

*A chi-square test was applied

All nurses had a mean BAI score of 11.0 ± 13.2. Among inpatient unit nurses, 54.2% were within the normal range for anxiety, 17.4% had mild anxiety, 14.2% had moderate anxiety, and 14.2% had severe anxiety. Among ICU nurses, 55.1% were normal, 22.5% had mild anxiety, 9.0% had moderate anxiety, and 13.5% had severe anxiety, with no significant difference between groups (*p* = 0.568). Also, no significant difference was observed in anxiety scores by work unit (*p* = 0.816) (Table 3).

**Table 3:** Department-wise comparison of anxiety levels among nurses in Diyarbakir - Diyarbakir, Türkiye, 2023.

		Inpatient		Intensive care		*p* ^*^
		*n*	%	*n*	%	
**Anxiety level**	Minimal	84	54.2	49	55.1	0.568
	Mild	27	17.4	20	22.5	
	Moderate	22	14.2	8	9.0	
	Severe	22	14.2	12	13.5	
**Anxiety score,Mean ± SD**		11.0 ± 13.2		10.8 ± 13.5		0.816^**^

*Chi-square test

**Mann”Whitney *U* test.

When comparing PETLDS scores by work unit, inpatient unit nurses (48.2 ± 20.0) and ICU nurses (45.6 ± 18.0) showed no significant differences in total or subscale scores (*p* > 0.05) (Table 4).

**Table 4:** Department-wise comparison of PETLDS scores among nurses in Diyarbakir - Diyarbakir, Türkiye, 2023.

	Inpatient	Intensive care	*p* ^*^
	Mean ± SD	Mean ± SD	
**Behavioral problems**	8.4 ± 4.2	8.0 ± 3.9	0.495
**Excitement limitation**	11.4 ± 5.5	10.9 ± 5.1	0.589
**Affective**	9.5 ± 4.0	9.4 ± 3.9	0.903
**Cognitive structure**	11.7 ± 5.1	11.0 ± 4.9	0.340
**Sleep problems**	7.1 ± 4.0	6.3 ± 3.5	0.144
**PETLDS total score**	48.2 ± 20.0	45.6 ± 18.0	0.407

*Mann”Whitney *U* test

PETLDS: Post-Earthquake Trauma Level Determination Scale

A significant positive correlation was observed between age and years of service and the Emotional subscale. Also, a significant positive correlation was found between years of service and the Emotional, Cognitive Structure, and Sleep Problems subscales, as well as the total PETLDS score. The anxiety score displayed a significant positive correlation with the PETLDS total score and all subscales. Furthermore, the subscales of the PETLDS were positively and significantly correlated with each other and with the total score (Table 5).

**Table 5:** Correlations between age, years of employment, anxiety scores, and post-earthquake trauma levels - Diyarbakir, Türkiye, 2023.

		Age	Years of employment	BAI score	Behavioral problems	Excitement limitation	Affective	Cognitive structure	Sleep problems
Years of employment	*r*	**.907**							
	*p*	**0.000**							
BAI score	*r*	.033	.055						
	*p*	0.604	0.392						
Behavioral problems	*r*	.020	.081	**.536**					
	*p*	0.756	0.210	**0.000**					
Excitement limitation	*r*	.052	.103	**.449**	**.723**				
	*p*	0.416	0.109	**0.000**	**0.000**				
Affective	*r*	**.161**	**.192**	**.374**	**.558**	**.625**			
	*p*	**0.012**	**0.003**	**0.000**	**0.000**	**0.000**			
Cognitive structure	*r*	.085	**.136**	**.515**	**.667**	**.687**	**.702**		
	*p*	0.184	**0.034**	**0.000**	**0.000**	**0.000**	**0.000**		
Sleep problems	*r*	.083	**.134**	**.570**	**.757**	**.701**	**.616**	**.759**	
	*p*	0.197	**0.036**	**0.000**	**0.000**	**0.000**	**0.000**	**0.000**	
PETLDS total score	*r*	.091	**.151**	**.557**	**.843**	**.871**	**.794**	**.896**	**.878**
	*p*	0.155	**0.018**	**0.000**	**0.000**	**0.000**	**0.000**	**0.000**	**0.000**

BAI: Beck Anxiety Inventory PETLDS: Post-Earthquake Trauma Level Determination Scale

## DISCUSSION

In this study, we examined the sociodemographic characteristics, earthquake experiences, anxiety levels, and PETLDS scores of inpatient unit and ICU nurses at Dicle University Hospital in Diyarbakır, Türkiye 5 months after the earthquake on February 6, 2023, in Türkiye. The results showed that both groups experienced a moderate level of trauma, with no statistically significant differences in any PETLDS subscale scores between them (*p* > 0.05).

The mean age and years of employment of inpatient unit nurses were significantly higher than those of ICU nurses (*p* < 0.001). Despite the lack of data on hospital policy, the aforementioned finding suggested that younger nurses might have been assigned to ICUs because ICUs often require overtime and are perceived as relatively more stressful than regular inpatient floors. A significant difference was also observed in marital status: 61.3% of inpatient unit nurses versus 48.3% of ICU nurses were married (*p* = 0.049), likely reflecting the younger age profile in intensive care. These findings suggested that professional experience and demographic factors may influence nurses’ placement within the hospital, thereby impacting their exposure to trauma and the development of traumatic stress symptoms.

A large majority of nurses (84.4%) in both groups had prior earthquake experience, underscoring the high seismic risk of the region. Likewise, most participants were located in one of the 10 affected provinces at the time of the earthquake (inpatient unit 95.5%; ICU 98.9%), indicating similar environmental exposure. The loss of a close relative during the earthquake was reported by 24.7% of ICU nurses compared with 17.4% of inpatient unit nurses, suggesting greater exposure to traumatic bereavement among the former and the possibility of deeper psychological effects.

BAI results revealed no significant difference in anxiety levels between inpatient unit and ICU nurses (*p* = 0.816). Minimal anxiety was observed in 54.2% of inpatient unit nurses and 55.1% of ICU nurses. The combined rates of mild and moderate anxiety were nearly identical (inpatient unit 31.6%; ICU 31.5%). Mild anxiety was slightly higher among ICU nurses (22.5%) and moderate anxiety was higher among inpatient unit nurses (17.4%). These differences might be attributable to the longer years of service among inpatient staff. Severe anxiety rates were similar in both groups. Overall, the anxiety levels were comparable across minimal, mild, moderate, and severe categories, with no significant intergroup differences.

No significant differences were observed between groups in terms of PETLDS total or subscale scores (*p* > 0.05). However, positive correlations between age and years of employment and several PETLDS subscales suggested that older, more experienced nurses might exhibit greater cumulative stress symptoms. Similarly, moderate anxiety was higher in the more experienced inpatient unit nurses. The observed positive correlation between anxiety scores and both PETLDS total and subscale scores supports the conclusion that post-earthquake anxiety is a key factor influencing traumatic stress symptoms^(24,25)^.

Previous studies have shown that major disasters such as earthquakes increase symptoms of depression, anxiety, and post-traumatic stress among healthcare workers^(24,25)^. Higher PETLDS scores have been reported among nurses indirectly impacted by an earthquake^(26)^. These studies also found that female sex, marriage, parenthood, and first-time earthquake exposure were associated with higher stress levels^(27)^. In contrast, our study found no significant difference in traumatic stress based on prior earthquake experience (*p* = 0.676). Numerous factors, including sex, professional and earthquake-related variables, personality traits, social support, coping strategies, resilience, and fulfillment of personal needs, have been shown to influence the mental health of post-earthquake healthcare workers, thereby highlighting the multidimensional nature of these influences.

One of the most common psychological conditions seen in healthcare workers after an earthquake is PTSD. One study found that 30% of healthcare staff working in the earthquake zone exhibited PTSD and 27.1% exhibited depression, compared with 10.2% and 9.7% in nonexposed staff, respectively, indicating the profound psychological impact of working in a disaster area^(28)^. Anxiety is another significant post-earthquake issue. A study examining the anxiety levels of emergency healthcare workers who worked in the aftermath of the Kahramanmaraş earthquake found that the anxiety levels were higher in healthcare workers who experienced the earthquake than in volunteer workers who moved to the earthquake region to assist^(29)^.

A study on survivors of the Wenchuan earthquake showed that PTSD prevalence decreased from 62.8% to 37.8% within the first 3 months^(30)^. These findings demonstrated that the passage of time after a disaster led to reductions in anxiety and trauma levels^(31)^. Our assessment 5 months after the earthquake suggested that traumatic stress levels might have begun to normalize over time.

However, many factors related to post-earthquake living conditions, such as home damage, the loss of loved ones, and personal injury, have been shown to elevate trauma levels^(32)^. In our sample, the relatively low proportions of moderate/severe home damage (7.78%) and injuries (3.27%) might explain the overall lower trauma and anxiety levels. Moreover, the high rate of prior earthquake experience (inpatient 85.2%; intensive care 83.1%) might have conferred some adaptive benefit. Available data indicate that healthcare workers directly or indirectly exposed to disaster zones may experience similar psychological impacts due to their professional roles, working environments, and individual coping mechanisms^(33)^.

We also observed a positive correlation between years of service and both the PETLDS total score and certain subscales. A previous study has suggested that longer tenure can be associated with increased anxiety and depressive symptoms^(34)^. Staff with longer service durations may experience greater occupational stress and strain; however, they may also develop more effective coping strategies. However, this relationship can be moderated by factors such as institutional support, ongoing training, and social interactions.

Our findings highlight the need to integrate structured psychological support within routine nursing operations in disaster-prone settings. Practical strategies may be as follows: periodic brief screening for anxiety and trauma symptoms with rapid, confidential referral; resilience-building and stress-management programs adapted to shift schedules; protected recovery time and micro-breaks during high-intensity shifts; peer-support networks and structured post-event debriefings; trauma-informed supervision; and targeted resources for subgroups with elevated risk indicators (e.g., longer tenure and cumulative stress, recent bereavement, or significant home damage).

From a management perspective, integrating mental health provisions into Hospital Disaster and Emergency Response Plans is essential. We recommend codifying a pre-/post-disaster mental health action plan with clear responsibilities, screening schedules, and referral workflows; conducting unit-level risk assessments to guide staffing, workload balancing, and resource allocation; training leaders in trauma-informed supervision and communication; monitoring the indicators of workforce well-being (e.g., absenteeism, turnover intention, and self-reported stress) with feedback loops to refine policies; and evaluating the feasibility and effectiveness of low-cost supports (e.g., brief debriefings and peer support) for scalability.

Finally, the 5-month interval since the earthquake likely influenced our findings. Trauma and stress symptoms can diminish over time, as individuals adapt, resume normal activities, utilize support resources, and process their experiences. This indicates that post-traumatic stress and anxiety levels can decrease over time as part of the psychological recovery process.

In summary, our study demonstrated that inpatient unit and ICU nurses experienced similar levels of trauma. However, long-term service, previous earthquake exposure, and individual psychological factors can complicate this relationship, suggesting the need for tailored support interventions.

## LIMITATIONS

This study had several limitations. Conducting the study at a single institution (Dicle University Hospital) might have limited the generalizability of the findings to nurses in other healthcare facilities or geographic regions affected by the earthquake. The cross-sectional design, which assessed trauma and anxiety levels at a single point in time, did not allow for causal inferences or for observing changes over time. Data collection occurred 5 months after the earthquake. As a result, some participants might have recovered or adapted to the situation. The lack of information on the pre-earthquake psychological status of nurses might also have restricted causal interpretations.

Although multivariable modeling can be informative, we intentionally did not perform adjusted regression analyses in this cross-sectional comparative study. In post-disaster contexts, several covariates are plausibly on the causal pathway or may act as colliders; routine adjustment can bias the target estimates. In line with our a priori comparative, noncausal objective and the small, nonsignificant unadjusted differences, we prioritized transparent unadjusted contrasts. Future multicenter and longitudinal studies, with prespecified minimal adjustment sets and temporally ordered measurements, are needed to provide robust adjusted estimates and evaluate the implementation and effectiveness of organizational mental health interventions in hospital settings.

## CONCLUSION

This study showed that both inpatient unit and ICU nurses had similar levels of post-earthquake trauma and anxiety. Although no significant differences emerged based on their work setting, it was clear that nurses might have experienced a certain degree of trauma and anxiety following an earthquake. Hence, it is concluded that healthcare workers themselves can be impacted by disasters or can be disaster survivors, and yet they must continue to provide care in the aftermath. Consequently, they require social, medical, and psychological support. In disaster settings, we should develop and implement measures to support the mental health of healthcare workers, such as psychological support programs, stress-management training, and professional peer-support networks. As essential members of the healthcare workforce, nurses’ psychological well-being is crucial for the sustainability of post-disaster healthcare services.

## Data Availability

The entire dataset supporting the results of this study is available upon request to the corresponding author.
