# Supplementary material for: Comparison of post-earthquake trauma and anxiety levels of intensive care unit nurses and inpatient unit nurses: a cross-sectional study
Source: Rev Esc Enferm USP. 2026 Mar 2;60:e20250312. doi: 10.1590/1980-220X-REEUSP-2025-0312en (PMC12952230; doi:10.1590/1980-220X-REEUSP-2025-0312en)
Supplement: Translation

# Translation: Comparación de los niveles de trauma y ansiedad tras el terremoto entre el personal de enfermería de la unidad de cuidados intensivos y el de la unidad de hospitalización: un estudio transversal

**DOI:** 10.1590/1980-220X-REEUSP-2025-0312es

**Authors:** Zehra Kilinç, Elif Ates Budak, Hatice Arslan, Süreyya Yiğitalp Rençber

**Affiliations:** 1 Dicle University, Faculty of Medicine, Department of Public Health, Diyarbakır, Turkey.; 2 Dicle University, Medical Faculty, Department of Psychiatry, Diyarbakir, Turkey.; 3 Dicle University, Hospital Health Care Services Assistant Manager, Diyarbakır, Turkey.; 4 Batman University, Faculty of Health Sciences, Department of Public Health, Batman, Turkey.

**Keywords:** Ansiedad, Terremotos, Hospitales, Personal de Enfermería, Trauma Psicológico

## Abstract

**Objetivo:**

Evaluar y comparar el trauma post-terremoto y la ansiedad en enfermeros de las unidades de cuidados intensivos y de hospitalización tras el terremoto de Kahramanmaraş ocurrido en febrero de 2023 en el Hospital Universitario de Dicle, en Diyarbakır, Turquía.

**Método:**

Se llevó a cabo un estudio transversal entre el 1 y el 31 de julio de 2023 con 244 enfermeros (89 de cuidados intensivos y 155 de unidades de hospitalización). Se aplicaron un formulario sociodemográfico, la Escala de Determinación del Nivel de Trauma Post-Terremoto (PETLDS) y el Inventario de Ansiedad de Beck (BAI). Los análisis estadísticos incluyeron las pruebas de chi cuadrado, *t* de Student, U de Mann-Whitney y la correlación de Spearman.

**Resultados:**

Del total de los participantes, el 12,3 % presentó ansiedad moderada y el 13,9 % presentó trauma grave. Las puntuaciones medias y las subescalas de la PETLDS no mostraron diferencias significativas entre ambos grupos (Hospitalización: 48,2 ± 20,0 vs. UCI: 45,6 ± 18,0; p > 0,05). Tampoco se observaron diferencias estadísticamente significativas en los niveles de ansiedad medidos a través del BAI según el tipo de unidad (p > 0,05). Sin embargo, ambos grupos presentaron proporciones relevantes de sintomatología psicológica.

**Conclusión:**

Tanto los enfermeros de cuidados intensivos como los de hospitalización experimentaron niveles comparables y considerables de trauma y ansiedad posteriores al desastre. Los hallazgos muestran la necesidad de integrar programas de apoyo psicológico y estrategias de resiliencia para el personal sanitario que trabaja durante y después de desastres.

## INTRODUCCIÓN

Los desastres naturales, como los terremotos, se encuentran entre los eventos incontrolables más devastadores, ya que causan impactos generalizados a nivel social y comunitario y provocan pérdidas significativas^(1,2)^. Un terremoto puede causar una gran pérdida de vidas y destrucción física, además de impactos sociales y psicológicos^(1,3)^. El miedo experimentado durante un terremoto, los daños en los hogares y lugares de trabajo y la pérdida de seres queridos pueden provocar estrés psicológico, lo que puede desencadenar trastornos psiquiátricos o exacerbar los síntomas existentes. Los problemas psicológicos posteriores a un terremoto suelen incluir ansiedad, depresión, ataques de pánico, trastornos del sueño y trastorno de estrés postraumático (TEPT)^(4)^.

En situaciones de desastre, los servicios de salud se enfrentan a retos que difieren notablemente de las operaciones rutinarias y que a menudo son impredecibles. Los hospitales deben seguir prestando atención a los pacientes, apoyo médico y servicios institucionales sin interrupción, mientras que los trabajadores de la salud desempeñan funciones vitales en la respuesta a emergencias y las intervenciones para salvar vidas^(5)^. Las enfermeras son el grupo profesional más numeroso dentro de la fuerza laboral continua que presta servicios de salud, y desempeñan un papel vital tanto en la fase de preparación como en la de rescate, contribuyendo así a la resiliencia de la comunidad ante los desastres^(6)^.

El entorno de trabajo y las características sociodemográficas de los profesionales de la atención de salud son factores importantes que influyen en su salud física y mental^(7)^. Las unidades de cuidados intensivos (UCI) y los servicios de urgencias se consideran áreas de trabajo de alto riesgo en los hospitales^(8)^. Los pacientes de la UCI a menudo se enfrentan a situaciones que ponen en peligro su vida, requieren ser monitoreados de manera estrecha y tienen tasas más altas de mortalidad y morbilidad; por lo tanto, las enfermeras de la UCI deben poseer habilidades y cualificaciones avanzadas^(9)^. La literatura muestra que, en circunstancias normales, las enfermeras de la UCI reportan niveles más altos de carga psicológica, como estrés y ansiedad, en comparación con las enfermeras de otras unidades^(10,11,12,13,14)^. Sin embargo, un estudio no encontró diferencias significativas en cuanto a depresión, ansiedad o factores estresantes en el lugar de trabajo entre el personal de enfermería de la UCI y el de las salas generales^(15)^.

La exposición del personal de enfermería de la UCI a acontecimientos traumáticos, como terremotos, ya sea presenciándolos, escuchando detalles sobre ellos o simplemente enterándose de ellos, puede desencadenar síntomas de TEPT, como revivir la experiencia, evitación, hiperactivación y cambios cognitivos^(16)^. Numerosos estudios han informado de un mayor riesgo de TEPT, depresión y ansiedad entre los servicios de emergencia y el personal de salud tras las catástrofes. Los datos disponibles también indican que la exposición crónica a un alto nivel de estrés en diversas unidades de enfermería está estrechamente relacionada con el absentismo y los problemas de salud, con la frecuente coexistencia de ansiedad y depresión^(17)^. Además, las enfermeras que trabajan bajo un estrés intenso o que son testigos de traumas suelen experimentar agotamiento, ansiedad, depresión y estrés traumático secundario^(18,19)^.

El 6 de febrero de 2023, dos grandes terremotos (Mw 7,7 y Mw 7,6) con epicentro en Kahramanmaraş causaron una destrucción generalizada y la pérdida de vidas humanas en 11 provincias de Turquía. Diyarbakır fue una de las ciudades afectadas, con 414 muertos, 902 heridos y daños graves en miles de edificios^(20)^. El objetivo de este estudio fue evaluar el impacto psicológico, concretamente los niveles de trauma y ansiedad, entre el personal de enfermería de la UCI y de la unidad de hospitalización general de un hospital universitario terciario de Diyarbakır cinco meses después de los terremotos^(21)^.

A pesar de las crecientes pruebas sobre la salud mental relacionada con los desastres entre los trabajadores de salud, siguen existiendo posibles lagunas de conocimiento en cuanto a las diferencias específicas de cada unidad en la sintomatología posterior al terremoto y las implicaciones prácticas para la gestión del personal hospitalario. Las pruebas son inconsistentes en cuanto a si las enfermeras de la UCI experimentan una mayor carga psicológica que las enfermeras de sala en contextos de desastre. Además, pocos estudios han investigado estas diferencias dentro de la misma institución y en el mismo intervalo de tiempo después del evento. Por otra parte, los estudios anteriores a menudo han subestimado la forma en que los resultados se traducen en la gestión de los recursos humanos y los programas de salud mental en el ámbito hospitalario.

Este estudio abordó estas lagunas comparando el trauma y la ansiedad tras el terremoto entre el personal de enfermería de la UCI y de la unidad de hospitalización de un hospital universitario terciario situado en una provincia afectada, cinco meses después de los terremotos de febrero de 2023. Se centró en dos grupos de gran relevancia que trabajan con políticas y recursos institucionales similares, proporcionando evidencia oportuna y orientada a la práctica para informar la planificación de recursos humanos, el desarrollo de la resiliencia y la elaboración de políticas de salud mental para el personal de enfermería en regiones propensas a desastres.

## MÉTODO

### Diseño del Estudio

Se trata de un estudio transversal elaborado de acuerdo con la lista de verificación Strengthening the Reporting of Observational Studies in Epidemiology (Fortalecimiento de la notificación de estudios observacionales en epidemiología).

### Población y Muestra

Este estudio transversal analítico se llevó a cabo del 1 al 31 de julio de 2023 en el Hospital Universitario Dicle de Diyarbakır, Turquía. La población objetivo estaba compuesta por las 450 enfermeras tituladas que trabajaban en las UCI para adultos y en las salas de hospitalización general durante el periodo de estudio. Se adoptó una estrategia de censo; se excluyó al personal de enfermería que estuvo de baja durante todo el periodo de reclutamiento. Se incluyó en el estudio a un total de 244 enfermeros (54,2 %) después de recibir información detallada sobre el objetivo del estudio y dar su consentimiento verbal.

### Herramientas de Recolección de Datos

Los datos se recopilaron en las propias unidades de enfermería mediante entrevistas presenciales utilizando un cuestionario estructurado en tres partes. La primera parte recopilaba las características sociodemográficas y las experiencias relacionadas con el terremoto. La segunda parte comprendía la Escala de Determinación del Nivel de Trauma Post-Terremoto (Post-Earthquake Trauma Level Determination Scale - PETLDS) de 20 ítems (escala Likert de 5 puntos, puntuación total de 20 a 100, α de Cronbach = 0,87 y umbral turco ≥ 52,4 ± 5,1 que indica un trauma clínicamente significativo). La tercera parte comprendía el Inventario de Ansiedad de Beck (Beck Anxiety Inventory - BAI) de 21 ítems (escala Likert de 4 puntos, puntuación total de 0 a 63 y α de Cronbach = 0,93, con puntuaciones interpretadas como: 0-7, ninguna; 8-15, leve; 16-25, moderada; y 26-63, ansiedad grave)^(22,23)^.

### Análisis Estadístico

Los resultados primarios fueron las puntuaciones totales del PETLDS y el BAI, mientras que la variable de exposición principal fue el entorno de trabajo (UCI frente a sala). Los posibles factores de confusión preespecificados incluyeron el sexo, la edad, el estado civil, el nivel educativo, los daños en el hogar relacionados con el terremoto, la experiencia previa de terremotos, la pérdida de un familiar o amigo cercano y las lesiones personales. Los análisis estadísticos se realizaron con IBM SPSS Statistics v24.

Las variables categóricas se presentaron como frecuencias y porcentajes, mientras que las variables continuas se expresaron como media ± desviación estándar o mediana [rango intercuartílico] según la prueba de normalidad de Kolmogorov-Smirnov. Las comparaciones entre grupos de datos categóricos, datos con distribución normal y datos con distribución no normal se realizaron utilizando la prueba χ^2^ de Pearson, la prueba *t* para muestras independientes y la prueba *U* de Mann-Whitney, respectivamente. Las correlaciones entre las variables continuas se examinaron con la correlación de Spearman. Los p-valores bilaterales <0,05 indicaron diferencias estadísticamente significativas. Dado el objetivo descriptivo y comparativo de este estudio transversal, los análisis primarios se centraron en comparaciones no ajustadas entre grupos (UCI frente a sala) sin inferencias causales. No ajustamos modelos de regresión multivariable porque varias covariables candidatas (por ejemplo, las pérdidas relacionadas con el terremoto) eran plausiblemente posteriores a la exposición (unidad de trabajo) en un entorno posterior al desastre; el ajuste rutinario de las variables posteriores puede introducir un sesgo al condicionar los mediadores o colisionadores. Además, las diferencias observadas entre los grupos eran pequeñas y no significativas, por lo que era poco probable que un ajuste adicional cambiara las conclusiones sustantivas, al tiempo que podría fomentar una interpretación excesiva. Hacemos hincapié en la estimación y la presentación de informes transparentes, y destacamos el modelado ajustado como una prioridad para futuras investigaciones multicéntricas y longitudinales.

### Aprobación Ética y Consentimiento

El protocolo del estudio fue aprobado por el Comité de Ética de Investigación Clínica No Intervencionista de la Universidad de Dicle (Decisión n.º 29, 14 de junio de 2023). Todos los procedimientos se ajustaron a la Declaración de Helsinki. El comité de ética aprobó el uso del consentimiento verbal informado para este estudio de riesgo mínimo basado en cuestionarios. Antes de participar, todas las enfermeras recibieron información estandarizada sobre el propósito, los procedimientos, los riesgos y los beneficios del estudio, y dieron su consentimiento verbal para participar. Se obtuvo el permiso administrativo para realizar el estudio dentro del hospital por parte de la dirección de la institución. Los datos se recolectaron de forma anónima, sin identificadores personales, y se almacenaron en dispositivos cifrados, a los que solo tenía acceso el equipo de investigación.

## RESULTADOS

El examen de las características demográficas y profesionales de las enfermeras participantes reveló que el 74,2 % de las enfermeras de la unidad de hospitalización regular y el 58,4 % de las enfermeras de la UCI eran mujeres. La edad media de las enfermeras de la unidad de hospitalización y de la UCI era de 37,0 ± 9,1 años y 31,7 ± 7,7 años, respectivamente. En cuanto a los años de servicio, las enfermeras de la unidad de hospitalización tenían una media de 15,0 ± 9,7 años, mientras que las enfermeras de la UCI tenían una media de 9,3 ± 8,0 años; esta diferencia fue estadísticamente significativa (p < 0,001). En cuanto al estado civil, el 61,3 % de las enfermeras de la unidad de hospitalización y el 48,3 % de las enfermeras de la UCI estaban casadas.

En cuanto al nivel educativo, el 60,6 % de las enfermeras de la unidad de hospitalización y el 52,8 % de las enfermeras de la UCI tenían una licenciatura (Tabla 1).

**Tabla 1:** Comparación de las características demográficas y profesionales de las enfermeras que trabajan en unidades de hospitalización y de cuidados intensivos - Diyarbakir, Turquía, 2023.

		Hospitalizados			Cuidados intensivos			*p* ^*^
		*n*		%	*n*		%
**Sexo**	Femenino	115		74,2	52		58,4	**0,011**
	Masculino	40		25,8	37		41,6	
**Edad, promedio ± DE**			37,0 ± 9,1			31,7 ± 7,7		< 0,001^**^
**Estado civil**	Soltero	60		38,7	46		51,7	**0,049**
	Casado	95		61,3	43		48,3	
**Nivel educativo**	Secundario	23		14,8	19		21,3	0,494
	Técnico	26		16,8	14		15,7	
	Profesional	94		60,6	47		52,8	
	Post graduado	12		7,7	9		10,1	
**Años de empleo, promedio ± DE**			15,0 ± 9,7			9,3 ± 8,0		*p* < 0,001^**^

*Prueba de chi cuadrado

**Prueba de Mann-Whitney *U*

Además, el 82,5 % de las enfermeras de unidades de hospitalización y el 83,1 % de las enfermeras de UCI habían experimentado anteriormente un terremoto, sin diferencias significativas entre los grupos (*p* = 0,676). Por otra parte, el 92,5 % de las enfermeras de unidades de hospitalización y el 98,9 % de las enfermeras de UCI trabajaban en una de las diez provincias afectadas por el terremoto en el momento del suceso, de nuevo sin diferencias significativas entre los grupos (p = 0,264). En cuanto a los daños en sus viviendas, el 53,5 % de las enfermeras de unidades de hospitalización no reportaron daños, el 38,1 % reportaron daños menores y el 8,4 % reportaron daños moderados a graves o derrumbes; entre las enfermeras de UCI, estas proporciones fueron del 61,8 %, 31,5 % y 6,7 %, respectivamente (p = 0,456). El 2,6 % de las enfermeras de la unidad de hospitalización y el 4,5 % de las enfermeras de la UCI informaron de lesiones sufridas durante el terremoto (p = 0,468). Por último, el 17,4 % de las enfermeras de la unidad de hospitalización y el 24,7 % de las enfermeras de la UCI perdieron a un familiar cercano en el terremoto, sin diferencias significativas entre los grupos (p = 0,171) (Tabla 2).

**Tabla 2:** Comparación por departamentos de las experiencias relacionadas con el terremoto entre el personal de enfermería de Diyarbakir, - Diyarbakir, Turquía, 2023.

		Hospitalizados		Cuidados intensivos		*p* ^*^
		*n*	%	*n*	%
¿Ha tenido la experiencia de un terremoto?	Si	132	85,2	74	83,1	0,676
	No	23	14,8	15	16,9	
¿Dónde estaba durante el terremoto?	Una de las diez ciudades más afectadas por el terremoto	148	95,5	88	98,9	0,264
	Otras ciudades	7	4,5	1	1,1	
¿Su casa tuvo daños por el terremoto?	Sin daños	83	53,5	55	61,8	0,456
	Pocos daños	59	38,1	28	31,5	
	Daños moderados o mayores/colapso	13	8,4	6	6,7	
¿**Tuvo alguna herida?**	Si	4	2,6	4	4,5	0,468
	No	151	97,4	85	95,5	
¿Perdió algún pariente por el terremoto?	Si	27	17,4	22	24,7	0,171

*Se aplicó una prueba de chi cuadrado

Todas las enfermeras obtuvieron una puntuación media en la escala BAI de 11,0 ± 13,2. Entre las enfermeras de la unidad de hospitalización, el 54,2 % se encontraba dentro del rango normal de ansiedad, el 17,4 % presentaba ansiedad leve, el 14,2 % ansiedad moderada y el 14,2 % ansiedad grave. Entre las enfermeras de la UCI, el 55,1 % se encontraba dentro de los límites normales, el 22,5 % presentaba ansiedad leve, el 9,0 % presentaba ansiedad moderada y el 13,5 % presentaba ansiedad grave, sin diferencias significativas entre los grupos (p = 0,568). Además, no se observaron diferencias significativas en las puntuaciones de ansiedad por unidad de trabajo (p = 0,816) (Tabla 3).

**Tabla 3:** Comparación por departamentos de los niveles de ansiedad entre el personal de enfermería en Diyarbakir - Diyarbakir, Turquía, 2023.

		Hospitalizados		Cuidados intensivos		*p* ^*^
		*n*	%	*n*	%
**Nivel de ansiedad**	Mínimo	84	54,2	49	55,1	0,568
	Leve	27	17,4	20	22,5	
	Moderado	22	14,2	8	9,0	
	Severo	22	14,2	12	13,5	
**Puntuación de ansiedad, Promedio ± DE**		11,0 ± 13,2		10,8 ± 13,5		0,816^**^

*Prueba de chi cuadrado

**Prueba de Mann-Whitney *U*.

Al comparar las puntuaciones PETLDS por unidad de trabajo, las enfermeras de la unidad de hospitalización (48,2 ± 20,0) y las enfermeras de la UCI (45,6 ± 18,0) no mostraron diferencias significativas en las puntuaciones totales o de las subescalas (*p* > 0,05) (Tabla 4).

**Tabla 4:** Comparación por departamentos de las puntuaciones PETLDS entre enfermeras en Diyarbakir - Diyarbakir, Turquía, 2023.

	Hospitalizados	Cuidados intensivos	*p* ^*^
	Promedio ± DE	Promedio ± DE	
Problemas de comportamiento	8,4 ± 4,2	8,0 ± 3,9	0,495
Limitación de emociones	11,4 ± 5,5	10,9 ± 5,1	0,589
Afectividad	9,5 ± 4,0	9,4 ± 3,9	0,903
Estructuras cognitivas	11,7 ± 5,1	11,0 ± 4,9	0,340
Problemas para dormir	7,1 ± 4,0	6,3 ± 3,5	0,144
Puntuación total PETLDS	48,2 ± 20,0	45,6 ± 18,0	0,407

*Prueba *U* de Mann-Whitney

PETLDS: Escala de determinación del nivel de trauma post-terremoto.

Se observó una correlación positiva significativa entre la edad y los años de servicio y la subescala Emocional. Además,se encontró una correlación positiva significativa entre los años de servicio y las subescalas Emocional, Estructura Cognitiva y Problemas de Sueño, así como la puntuación total del PETLDS. La puntuación de ansiedad mostró una correlación positiva significativa con la puntuación total del PETLDS y todas las subescalas. Además, las subescalas del PETLDS se correlacionaron de manera positiva y significativa entre sí y con la puntuación total (Tabla 5).

**Tabla 5:** Correlaciones entre la edad, los años de empleo, las puntuaciones de ansiedad y los niveles de trauma post-terremoto - Diyarbakir, Turquía, 2023.

		Edad	Años de empleo	Puntuación BAI	Problemas de comportamiento	Limitación de emociones	Afectividad	Estructura cognitiva	Problemas para dormir
Años de empleo	*r*	**,907**							
	*p*	**0,000**							
Puntuación BAI	*r*	,033	,055						
	*p*	0,604	0,392						
Problemas de comportamiento	*r*	,020	,081	**,536**					
	*p*	0,756	0,210	**0,000**					
Limitación de emociones	*r*	,052	,103	**,449**	**,723**				
	*p*	0,416	0,109	**0,000**	**0,000**				
Afectividad	*r*	**,161**	**,192**	**,374**	**,558**	**,625**			
	*p*	**0,012**	**0,003**	**0,000**	**0,000**	**0,000**			
Estructura cognitiva	*r*	,085	**,136**	**,515**	**,667**	**,687**	**,702**		
	*p*	0,184	**0,034**	**0,000**	**0,000**	**0,000**	**0,000**		
Problemas para dormir	*r*	,083	**,134**	**,570**	**,757**	**,701**	**,616**	**,759**	
	*p*	0,197	**0,036**	**0,000**	**0,000**	**0,000**	**0,000**	**0,000**	
Puntuación total PETLDS	*r*	,091	**,151**	**,557**	**,843**	**,871**	**,794**	**,896**	**,878**
	*p*	0,155	**0,018**	**0,000**	**0,000**	**0,000**	**0,000**	**0,000**	**0,000**

BAI: (Beck Anxiety Inventory) Inventario de Ansiedad de Beck PETLDS: (Post-Earthquake Trauma Level Determination Scale) Escala de Determinación del Nivel de Trauma Post-Terremoto

## DISCUSIÓN

En este estudio, examinamos las características sociodemográficas, las experiencias del terremoto, los niveles de ansiedad y las puntuaciones PETLDS de las enfermeras de la unidad de hospitalización y de la UCI del Hospital Universitario Dicle de Diyarbakır, Turquía, cinco meses después del terremoto del 6 de febrero de 2023 en Turquía.

Los resultados mostraron que ambos grupos experimentaron un nivel moderado de trauma, sin diferencias estadísticamente significativas en ninguna de las puntuaciones de la subescala PETLDS entre ellos (p > 0,05).

La edad media y los años de empleo de las enfermeras de la unidad de hospitalización fueron significativamente superiores a los de las enfermeras de la UCI (p < 0,001). A pesar de la falta de datos sobre la política del hospital, el hallazgo mencionado sugiere que las enfermeras más jóvenes podrían haber sido asignadas a las UCI porque estas suelen requerir horas extras y se perciben como relativamente más estresantes que las plantas de hospitalización habituales. También se observó una diferencia significativa en el estado civil: el 61,3 % de las enfermeras de la unidad de hospitalización frente al 48,3 % de las enfermeras de la UCI estaban casadas (p = 0,049), lo que probablemente refleja el perfil de edad más joven en cuidados intensivos. Estos hallazgos sugieren que la experiencia profesional y los factores demográficos pueden influir en la ubicación de las enfermeras dentro del hospital, lo que repercute en su exposición al trauma y en el desarrollo de síntomas de estrés traumático.

La gran mayoría de las enfermeras (84,4 %) de ambos grupos había vivido anteriormente un terremoto, lo que pone de relieve el alto riesgo sísmico de la región. Del mismo modo, la mayoría de las participantes se encontraba en una de las diez provincias afectadas en el momento del terremoto (unidad de hospitalización 95,5 %; 98,9 % en la UCI), lo que indica una exposición ambiental similar. El 24,7 % de las enfermeras de la UCI informó haber perdido a un familiar cercano durante el terremoto, en comparación con el 17,4 % de las enfermeras de la unidad de hospitalización, lo que sugiere una mayor exposición al duelo traumático entre las primeras y la posibilidad de efectos psicológicos más profundos.

Los resultados de la BAI no revelaron diferencias significativas en los niveles de ansiedad entre las enfermeras de la unidad de hospitalización y las de la UCI (*p* = 0,816). Se observó una ansiedad mínima en el 54,2 % de las enfermeras de la unidad de hospitalización y en el 55,1 % de las enfermeras de la UCI. Las tasas combinadas de ansiedad leve y moderada fueron casi idénticas (unidad de hospitalización 31,6 %; UCI 31,5 %). La ansiedad leve fue ligeramente superior entre las enfermeras de la UCI (22,5 %) y la ansiedad moderada fue superior entre las enfermeras de la unidad de hospitalización (17,4 %). Estas diferencias podrían atribuirse a los más años de servicio del personal de hospitalización. Las tasas de ansiedad grave fueron similares en ambos grupos. En general, los niveles de ansiedad fueron comparables en las categorías mínima, leve, moderada y grave, sin diferencias significativas entre los grupos.

No se observaron diferencias significativas entre los grupos en cuanto a las puntuaciones totales o de las subescalas del PETLDS (p > 0,05). Sin embargo, las correlaciones positivas entre la edad y los años de empleo y varias subescalas del PETLDS sugirieron que las enfermeras de más edad y con más experiencia podrían presentar mayores síntomas de estrés acumulado. Del mismo modo, la ansiedad moderada fue mayor entre las enfermeras de la unidad de hospitalización con más experiencia. La correlación positiva observada entre las puntuaciones de ansiedad y las puntuaciones totales y de las subescalas del PETLDS respalda la conclusión de que la ansiedad posterior al terremoto es un factor clave que influye en los síntomas de estrés traumático^(24,25)^.

Estudios anteriores han demostrado que los grandes desastres, como los terremotos, aumentan los síntomas de depresión, ansiedad y estrés postraumático entre los trabajadores de salud (24,25). Se han registrado puntuaciones más altas en la PETLDS entre las enfermeras afectadas indirectamente por un terremoto^(26)^. Estos estudios también revelaron que el sexo femenino, el matrimonio, la maternidad y la exposición a un terremoto por primera vez se asociaban con niveles de estrés más elevados^(27)^. Por el contrario, nuestro estudio no encontró diferencias significativas en el estrés traumático en función de la experiencia previa con terremotos (p = 0,676). Se ha demostrado que numerosos factores, entre ellos el sexo, las variables profesionales y relacionadas con los terremotos, los rasgos de personalidad, el apoyo social, las estrategias de afrontamiento, la resiliencia y la satisfacción de las necesidades personales, influyen en la salud mental de los trabajadores de salud después de un terremoto, lo que pone de relieve la naturaleza multidimensional de estas influencias.

Una de las afecciones psicológicas más comunes que se observan en los trabajadores de salud después de un terremoto es el PETLDS. Un estudio reveló que el 30 % del personal de salud que trabajaba en la zona del terremoto presentaba PETLDS y el 27,1 % presentaba depresión, en comparación con el 10,2 % y el 9,7 % del personal no expuesto, respectivamente, lo que indica el profundo impacto psicológico de trabajar en una zona de desastre^(28)^. La ansiedad es otro problema importante tras un terremoto. Un estudio que examinó los niveles de ansiedad de los trabajadores de salud de urgencias que trabajaron tras el terremoto de Kahramanmaraş reveló que los niveles de ansiedad eran más altos en los trabajadores de salud que vivieron el terremoto que en los voluntarios que se trasladaron a la región afectada para ayudar^(29)^.

Un estudio sobre los sobrevivientes del terremoto de Wenchuan mostró que la prevalencia del trastorno de estrés postraumático disminuyó del 62,8 % al 37,8 % en los primeros tres meses^(30)^. Estos hallazgos demostraron que el paso del tiempo después de un desastre condujo a una reducción de los niveles de ansiedad y trauma^(31)^. Nuestra evaluación cinco meses después del terremoto sugirió que los niveles de estrés traumático podrían haber comenzado a normalizarse con el tiempo.

Sin embargo, se ha demostrado que muchos factores relacionados con las condiciones de vida tras el terremoto, como los daños en las viviendas, la pérdida de seres queridos y las lesiones personales, elevan los niveles de trauma^(32)^. En nuestra muestra, las proporciones relativamente bajas de daños moderados/graves en las viviendas (7,78 %) y lesiones (3,27 %) podrían explicar los niveles generales más bajos de trauma y ansiedad. Además, la alta tasa de experiencia previa en terremotos (pacientes hospitalizados 85,2 %; cuidados intensivos 83,1 %) podría haber conferido algún beneficio adaptativo. Los datos disponibles indican que los trabajadores de salud expuestos directa o indirectamente a zonas de desastre pueden experimentar impactos psicológicos similares debido a sus funciones profesionales, entornos de trabajo y mecanismos de afrontamiento individuales^(33)^.

También observamos una correlación positiva entre los años de servicio y la puntuación total del PETLDS y ciertas subescalas. Un estudio anterior ha sugerido que una antigüedad mayor puede estar asociada con un aumento de los síntomas de ansiedad y depresión^(34)^. El personal con una antigüedad mayor puede experimentar un mayor estrés y tensión ocupacionales; sin embargo, también puede desarrollar estrategias de afrontamiento más eficaces. No obstante, esta relación puede verse moderada por factores como el apoyo institucional, la formación continua y las interacciones sociales.

Nuestros hallazgos ponen de relieve la necesidad de integrar el apoyo psicológico estructurado en las operaciones rutinarias de enfermería en entornos propensos a desastres. Las estrategias prácticas pueden ser las siguientes: evaluación periódica breve de los síntomas de ansiedad y trauma con derivación rápida y confidencial; programas de desarrollo de la resiliencia y gestión del estrés adaptados a los turnos de trabajo; tiempo de recuperación protegido y microdescansos durante los turnos de alta intensidad; redes de apoyo entre compañeros y sesiones informativas estructuradas después de los eventos; supervisión informada sobre el trauma; y recursos específicos para subgrupos con indicadores de riesgo elevados (por ejemplo, antigüedad y estrés acumulado, duelo reciente o daños importantes en el hogar)..

Desde el punto de vista de la gestión, es esencial integrar las disposiciones en materia de salud mental en los planes de respuesta a desastres y emergencias de los hospitales. Recomendamos codificar un plan de acción de salud mental antes y después de un desastre con responsabilidades claras, calendarios de detección y flujos de trabajo de derivación; realizar evaluaciones de riesgo a nivel de unidad para orientar la dotación de personal, el equilibrio de la carga de trabajo y la asignación de recursos; formar a los líderes en supervisión y comunicación informadas sobre el trauma; monitorear los indicadores del bienestar de la plantilla (por ejemplo, absentismo, intención de rotación y estrés autoinformado) con bucles de retroalimentación para perfeccionar las políticas; y evaluar la viabilidad y la eficacia de los apoyos de bajo costo (por ejemplo, breves sesiones informativas y apoyo entre compañeros) para su escalabilidad.

Por último, es probable que el intervalo de cinco meses transcurrido desde el terremoto haya influido en nuestros resultados. Los síntomas de trauma y estrés pueden disminuir con el tiempo, a medida que las personas se adaptan, reanudan sus actividades normales, utilizan los recursos de apoyo y procesan sus experiencias. Esto indica que los niveles de estrés postraumático y ansiedad pueden disminuir con el tiempo como parte del proceso de recuperación psicológica.

En resumen, nuestro estudio demostró que las enfermeras de la unidad de hospitalización y de la UCI experimentaron niveles similares de trauma. Sin embargo, el servicio a largo plazo, la exposición previa a terremotos y los factores psicológicos individuales pueden complicar esta relación, lo que sugiere la necesidad de intervenciones de apoyo personalizadas.

## LIMITACIONES

Este estudio tuvo varias limitaciones. La realización del estudio en una sola institución (el Hospital Universitario de Dicle) podría haber limitado la generalización de los resultados a las enfermeras de otros centros de salud o regiones geográficas afectadas por el terremoto. El diseño transversal, que evaluó los niveles de trauma y ansiedad en un solo momento, no permitió realizar inferencias causales ni observar cambios a lo largo del tiempo. La recolección de datos se realizó cinco meses después del terremoto.

Como resultado, es posible que algunos participantes se hubieran recuperado o adaptado a la situación. La falta de información sobre el estado psicológico de las enfermeras antes del terremoto también podría haber restringido las interpretaciones causales. Aunque los modelos multivariables pueden ser informativos, en este estudio comparativo transversal no realizamos intencionadamente análisis de regresión ajustados. En contextos posteriores a desastres, es plausible que varias covariables se encuentren en la vía causal o actúen como colisionadores; el ajuste rutinario puede sesgar las estimaciones objetivo. En línea con nuestro objetivo comparativo a priori, no causal, y con las pequeñas diferencias no significativas sin ajustar, dimos prioridad a los contrastes transparentes sin ajustar. Se necesitan futuros estudios multicéntricos y longitudinales, con conjuntos de ajustes mínimos preespecificados y mediciones ordenadas temporalmente, para proporcionar estimaciones ajustadas sólidas y evaluar la implementación y la eficacia de las intervenciones organizativas de salud mental en entornos hospitalarios.

## CONCLUSIÓN

Este estudio demostró que tanto el personal de enfermería de las unidades de hospitalización como el de las UCI presentaban niveles similares de trauma y ansiedad tras el terremoto. Aunque no se observaron diferencias significativas en función de su entorno de trabajo, quedó claro que el personal de enfermería podría haber experimentado cierto grado de trauma y ansiedad tras el terremoto. Por lo tanto, se concluye que los propios trabajadores de salud pueden verse afectados por las catástrofes o ser supervivientes de las mismas, y sin embargo deben seguir prestando asistencia tras ellas. En consecuencia, necesitan apoyo social, médico y psicológico. En situaciones de desastre, debemos desarrollar y aplicar medidas para apoyar la salud mental de los trabajadores de salud, como programas de apoyo psicológico, formación en gestión del estrés y redes profesionales de apoyo entre compañeros. Como miembros esenciales del personal de salud, el bienestar psicológico de las enfermeras es crucial para la sostenibilidad de los servicios de salud después de un desastre.

## Data Availability

El conjunto de datos que respaldan las conclusiones de este estudio está disponible previa solicitud al autor correspondiente.
